# Quantification and visualization of cardiovascular 4D velocity mapping accelerated with parallel imaging or k-t BLAST: head to head comparison and validation at 1.5 T and 3 T

**DOI:** 10.1186/1532-429X-13-55

**Published:** 2011-10-04

**Authors:** Marcus Carlsson, Johannes Töger, Mikael Kanski, Karin Markenroth Bloch, Freddy Ståhlberg, Einar Heiberg, Håkan Arheden

**Affiliations:** 1 Dept. of Clinical Physiology, Lund University and Skane University Hospital, Lund, Sweden; 2 Philips Healthcare, Lund, Sweden; 3 Dept. of Radiation Physics, Lund University, Lund, Sweden

## Abstract

**Background:**

Three-dimensional time-resolved (4D) phase-contrast (PC) CMR can visualize and quantify cardiovascular flow but is hampered by long acquisition times. Acceleration with SENSE or k-t BLAST are two possibilities but results on validation are lacking, especially at 3 T. The aim of this study was therefore to validate quantitative in vivo cardiac 4D-acquisitions accelerated with parallel imaging and k-t BLAST at 1.5 T and 3 T with 2D-flow as the reference and to investigate if field strengths and type of acceleration have major effects on intracardiac flow visualization.

**Methods:**

The local ethical committee approved the study. 13 healthy volunteers were scanned at both 1.5 T and 3 T in random order with 2D-flow of the aorta and main pulmonary artery and two 4D-flow sequences of the heart accelerated with SENSE and k-t BLAST respectively. 2D-image planes were reconstructed at the aortic and pulmonary outflow. Flow curves were calculated and peak flows and stroke volumes (SV) compared to the results from 2D-flow acquisitions. Intra-cardiac flow was visualized using particle tracing and image quality based on the flow patterns of the particles was graded using a four-point scale.

**Results:**

Good accuracy of SV quantification was found using 3 T 4D-SENSE (r^2 ^= 0.86, -0.7 ± 7.6%) and although a larger bias was found on 1.5 T (r^2 ^= 0.71, -3.6 ± 14.8%), the difference was not significant (p = 0.46). Accuracy of 4D k-t BLAST for SV was lower (p < 0.01) on 1.5 T (r^2 ^= 0.65, -15.6 ± 13.7%) compared to 3 T (r^2 ^= 0.64, -4.6 ± 10.0%). Peak flow was lower with 4D-SENSE at both 3 T and 1.5 T compared to 2D-flow (p < 0.01) and even lower with 4D k-t BLAST at both scanners (p < 0.01). Intracardiac flow visualization did not differ between 1.5 T and 3 T (p = 0.09) or between 4D-SENSE or 4D k-t BLAST (p = 0.85).

**Conclusions:**

The present study showed that quantitative 4D flow accelerated with SENSE has good accuracy at 3 T and compares favourably to 1.5 T. 4D flow accelerated with k-t BLAST underestimate flow velocities and thereby yield too high bias for intra-cardiac quantitative in vivo use at the present time. For intra-cardiac 4D-flow visualization, however, 1.5 T and 3 T as well as SENSE or k-t BLAST can be used with similar quality.

## Background

Blood flow quantified by two-dimensional velocity encoded phase contrast (PC) CMR [1] is an important part of the SCMR recommended scan protocol for valvular disease and patients with congenital heart disease [2]. The accuracy is high when using a non-segmented approach [3,4] but several acquisitions are often required when assessing a patient and the newer segmented breath-hold sequences have showed lower accuracy [5,6]. Three-dimensional time-resolved (4D) PC CMR can be used to visualize and quantify cardiovascular flow and the desired imaging planes can be reconstructed after the acquisition [7]. The scanning time is long when acquiring the entire heart (20-40 minutes) even if standard acceleration techniques such as parallel imaging are used [7]. This hampers the clinical use of the technique but also the application of intra-cardiac 4D-flow in research studies of patients who may not tolerate lengthy scanning times.

k-t BLAST (Broad-use Linear Speed-up technique) is a method employed to reduces scan time using under-sampling of data in k-t space [8] and this technique has been used to accelerate 2D-flow measurements in vivo [9,10]. The 4D-flow application for k-t BLAST was described in a phantom by Marshall [11] and recently Stadlbauer et al found limitations with temporal blurring when comparing k-t BLAST with SENSE acceleration for 4D aortic flow measurements at 1.5 T [12]. However, 4D k-t BLAST has not been used or validated for intra-cardiac acquisitions. Most quantitative studies in vivo of 4D flow to date have used 1.5 T [13] but higher field strength have inherent benefits such as reduced noise and improved image quality [7]. This has been shown for 2D-flow acquisitions [14] but not for 4D-flow.

Therefore, the aim of this study was to validate quantitative in vivo cardiac 4D-flow measurements accelerated with parallel imaging and k-t BLAST at 1.5 T and 3 T with 2D-flow as the reference. Also, we aimed to investigate if intra-cardiac flow visualization differed with SENSE compared to k-t BLAST and on 1.5 T compared to 3 T.

## Methods

### Study design

The local ethical committee approved the study and informed consent was obtained from each volunteer. Cardiac 4D-flow with k-t BLAST and parallel imaging (SENSE) and 2D-flow measurements of the aorta and pulmonary trunk were obtained from 13 healthy volunteers (32 ± 12 years, 9 males). All volunteers were scanned in both a 1.5 T and 3 T Philips Achieva (Philips Medical Systems, Best, the Netherlands) on the same day in random order.

### MRI-sequence parameters

4D-flow-k-t BLAST: A turbo field echo (TFE) sequence with prospective ECG-triggering and k-t BLAST speed-up factor of 5 was used. Typical imaging parameters were: TE/TR/flip: 3.7/7.6 ms/8°, 15 time phases acquired and voxel size 3 × 3 × 3 mm^3^. The k-t BLAST net acceleration factor was 4.1-4.5 (5-fold acceleration with 11 lines of training data in both the ky and kz directions). The regularization matrix was calculated from the training data, and no additional terms were added. A segmentation factor of 2 was possible in subjects with heart rate below 70/min. The temporal resolution varied from 45-60 ms. The acquisition times for 4D-k-t BLAST were 10.8 ± 0.7 min (range 8-14 min). In six scans reconstruction using k-t SENSE was performed and in these cases a regularization factor of 0.5 was used. These data were analysed together with the k-t BLAST data, and are thus included in results labeled k-t BLAST.

4D-flow-SENSE: A turbo field echo (TFE) sequence with retrospective ECG-triggering and respiratory navigator with a segmentation factor of 2 and SENSE parallel imaging factor of 2 was used [15]. Typical imaging parameters were: TE/TR/flip: 3.7/6.3 ms/8°. Number of time phases acquired was dependent on heart rate and set to the maximum with a preserved segmentation factor of 2. The acquired temporal resolution varied from 50-55 ms, i.e. 14-22 phases acquired and thereafter reconstructed to 40 time phases. Voxel size was 3 × 3 × 3 mm^3^. The mean matrix size was 83 × 83 × 48 and the acquisition times for 4D-SENSE were 22.5 ± 0.3 min (range 14-33 min).

2D-flow was acquired with a non-segmented PC-FFE sequence with retrospective ECG-triggering and no respiratory navigor. Typical imaging parameters were: TE/TR/flip: 5.3/8.6 ms/15°, 35 time phases and voxel size 1.2 × 1.2 × 6 mm^3^.

Cine images were obtained using a steady state free precession (ssfp) sequence with retrospective ECG-triggering. Typical imaging parameters were: TE/TR: 2.8/1.4 ms, α:60°, in-plane spatial resolution 1.3 × 1.3 mm; slice thickness 8.0 mm, no gap; temporal resolution 30 ms.

### Image analysis

Concomitant gradients were compensated for by the MR scanner software. We developed a new module to the imaging software Segment (http://segment.heiberg.se) [16] for analysis of the 4D-flow images. This module involves a first-order phase background correction, phase unwrapping and the possibility to reconstruct the 4D-dataset into any 2D plane or 3D imaging stack. Thereby, 2D-images perpendicular to the aorta and pulmonary artery were reconstructed from the 4D-flow data in the identical imaging plane as the 2D-flow was acquired. Quantitative flow can be measured in both the original 2D-images and the derived 2D-images from the 4D-dataset. Semi-automatic outlining of the aorta and pulmonary trunk in the velocity encoded 2D-images were performed and the contours were transferred to the reconstructed 4D-images and manually corrected when needed. Stroke volume (SV) was calculated by integrating the flow curve over the entire cardiac cycle. Signal to noise ratio was calculated in k-t BLAST images using a region of interest (ROI) in the aorta and pulmonary trunk for signal (ROI_vessel_) and an ROI outside the subject for noise (ROI_outside_). SNR was calculated as 0.655 × (mean signal intensitity ROI_vessel_)/(standard deviation of noise ROI_outside_) [17].

### Quality assessment of flow visualization

Ensight 9.1 (CEI, USA) was used for flow visualization of intracardiac blood flow using particle tracing. Particle emitters were placed in all four chambers, and particles were released every 10 ms over the full cardiac cycle. One blinded observer graded the quality of the data based on the requirement that particles stay in the blood pool [18] as defined by cine images. Images were graded according to the following scale: 0) ideal, 1) only few particles leaving the blood pool, 2) moderate amount of particles leaving the blood pool and 3) large amount of particles leaving the blood pool rendering the images unusable. This grading was performed for the four emitters and the mean quality score was calculated for each subject.

### Statistical analysis

All values are given as mean ± SD. Stroke volumes (SV) and peak flows obtained from different sequences and scanners were compared using a paired non-parametric two-tailed test (Wilcoxon) and linear regression. Bias between SV measurements and peak flow were calculated according to Bland-Altman analysis (mean ± SD). Flow visualization mean quality scores were compared using a paired non-parametric two-tailed test (Wilcoxon). Results with a p-value < 0.05 were considered statistically significant.

## Results

### Two-dimensional flow measurements

**Figure 1:**
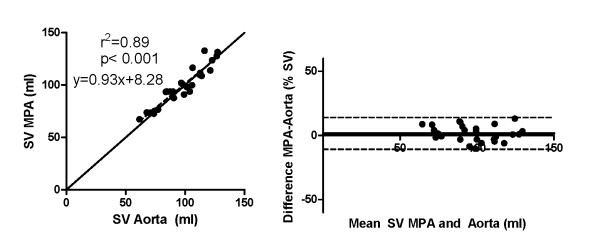
**There was a strong correlation between stroke volumes (SV) of the aorta and main pulmonary artery (MPA) with 2D-flow (left, solid line represents line of identity)**. Bias was low (1.7 ± 6.3%, right).

2D-flow measurements of the aorta and main pulmonary artery showed a strong correlation (r^2 ^= 0.89, y = 0.93x+8.28, p < 0.001) and low bias (1.7 ± 6.3%), Figure 1. The QP/QS for the subjects at 1.5 and 3 T were 1.03 ± 0.09. No significant differences were found when comparing stroke volumes between the two field strengths (p = 0.59) or QP/QS (p = 0.68).

### 4D flow curves and peak flow

**Figure 2:**
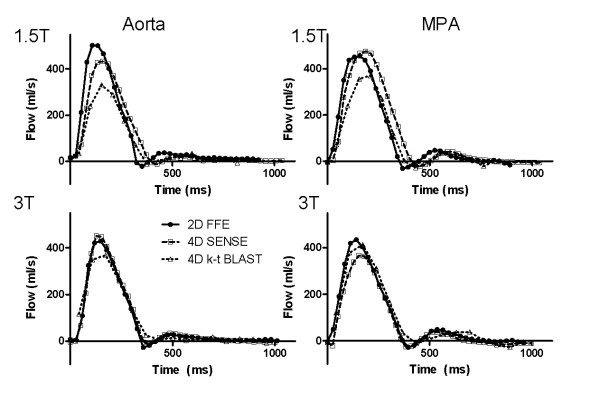
**Typical flow graphs over the cardiac cycle in one subject at 1.5 T (top row) and 3 T (bottom row) for the aorta (left column) and main pulmonary artery (MPA, right column)**. Remaining subjects are shown in the Additional files.

One k-t BLAST 4D data set at 3 T was excluded because of suboptimal image quality. Figure 2 shows the flow curves over the cardiac cycle for 4D-SENSE, k-t BLAST and 2D-flow for one subject. Flow curves for six additional subjects are shown in Additional file 1 and 2. The 4D-SENSE flow curves are similar to the 2D-flow (Figure 2) but peak flow was lower on both 1.5 T and 3 T (p < 0. 01 for both), (Table 1). Peak flow on 4D k-t BLAST was lower both compared to 2D flow and 4D-SENSE (p < 0.001 for both) on 1.5 T (Table 1). Similarly, on 3 T peak flow with 4D k-t BLAST was lower both compared to 2D flow (p < 0.001) and 4D-SENSE (p < 0. 01). SNR was 2.2 ± 0.9 times higher on 3 T compared to 1.5 T (p = 0.02)

**Table 1:** Bias and r^2 ^for stroke volume (SV) and peak flows obtained with 4D flow sequences compared with 2D-flow.

Sequence	r^2^	SV, bias ± SD	Peak flow ml/s (mean ± SD)
1.5 T SENSE = 2	0.71	-3.6 ± 14.8%	402 ± 86**

3 T SENSE = 2	0.86	-0.7 ± 7.6%	421 ± 75**

1.5 T k-t BLAST = 5	0.65	-15.6 ± 13.7%	353 ± 77***

3 T k-t BLAST = 5	0.64	-4.6 ± 10.0%	389 ± 68***

Peak flow with 2D-flow at 1.5 T: 439 ± 86 ml/s and 3 T: 448 ± 83 ml/s. ** p < 0.01 and *** p < 0.001 compared to peak flow on 2D-flow.

### 4D flow for stroke volume

**Figure 3:**
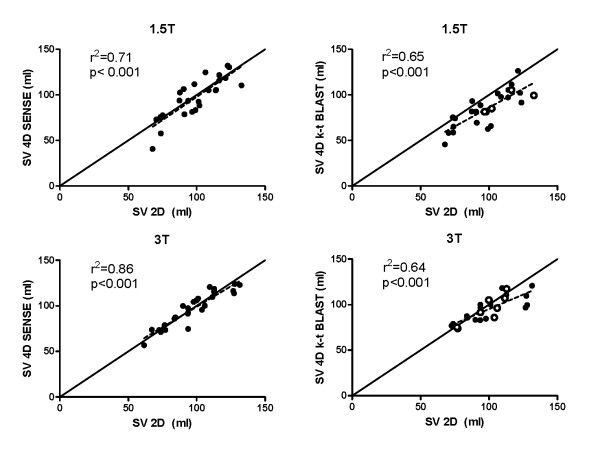
**The correlation of stroke volumes (SV) with 4D-SENSE flow acquisitions and 2D flow (left panels) was higher for acquisitions on 3 T (bottom) compared to 1.5 T (top)**. The correlations for k-t BLAST (right panels) on 3 T and 1.5 T were similar. Solid line represents line of identity. Results from k-t SENSE are shown as white circles.

**Figure 4:**
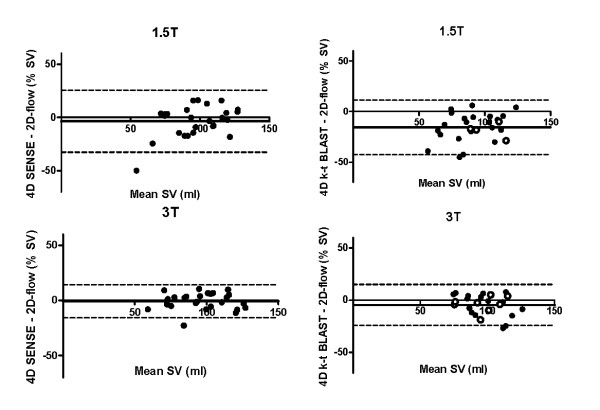
**Bland-Altman analysis of stroke volume (SV) quantified on 4D flow acquisitions and 2D flow acquisitions on both 1.5 T (top row) and 3 T (bottom row) accelerated with SENSE (left panels) and k-t BLAST (right panels)**. Results from k-t SENSE are shown as white circles.

Stroke volume on 4D-SENSE (96.2 ± 22.6 ml) and 2D (98.4 ± 18.7 ml) did not differ significantly on 1.5 T (p = 0.45) and regression analysis showed a strong correlation (r^2 ^= 0.71, Figure 3 and Table 1). However, the bias was -3.6 ± 14.8% (Figure 4). There was no significant difference in SV on 3 T (p = 0.86) with 4D-SENSE (96.7 ± 18.9 ml) compared to 2D (97.3 ± 19.5 ml), (Figure 4). Regression analysis showed a higher r^2 ^(r^2 ^= 0.86) and lower bias on 3 T (-0.7 ± 7.6%) compared to 1.5 T but the difference was not significant (p = 0.46). SV quantified using 4D k-t BLAST was lower compared to 2D on 1.5 T (84.8 ± 19.1 ml and 98.4 ± 18.7 ml, respectively, p < 0.001). On 3 T there was no significant difference on SV quantified using 4D k-t BLAST (95.2 ± 14.0 ml) and 2D (97.3 ± 19.5 ml, p = 0.10). Regression analysis and Bland-Altman analysis for SV quantified with k-t BLAST compared to 2D-flow showed higher agreement on 3 T (r^2 ^= 0.64, -4.6 ± 10.0%) compared to 1.5 T (r^2 ^= 0.65, -15.6 ± 13.7%, p < 0.01), Figures 3 and 4.

### Quality assessment of flow visualization

**Figure 5:**
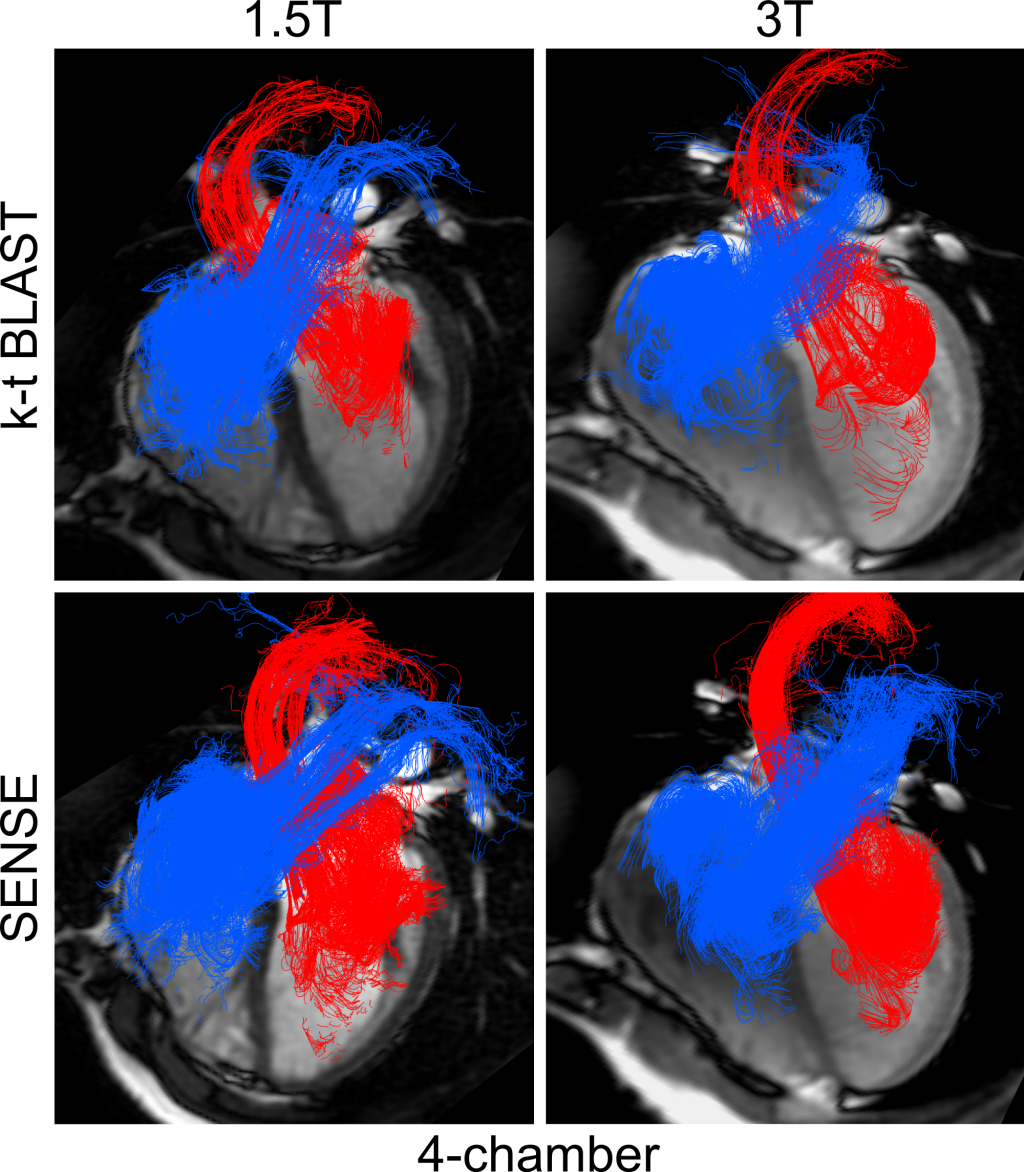
**Visualization of intra-cardiac 4D-flow using particle tracing with four-chamber cine images for anatomical reference, in the same subject as Figure 6**. Flow is coloured blue on the right side and red on the left side of the heart. Only a small amount of particles exhibit a non-physiological flow, which was read as good image quality. No major differences were seen between 1.5 T or 3 T nor between k-t BLAST or SENSE.

**Figure 6:**
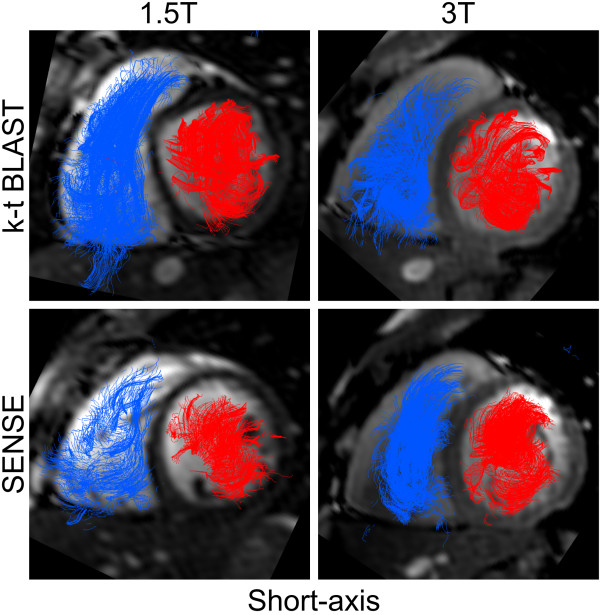
**Visualization of intra-cardiac 4D-flow using particle tracing with short-axis cine images for anatomical reference in the same subject as Figure 5**. Flow is coloured blue on the right side and red on the left side of the heart. Only a small amount of particles exhibit a non-physiological flow, which was read as good image quality. No major differences were seen between 1.5 T or 3 T nor between k-t BLAST or SENSE.

Examples of flow visualizations are shown in Figure 5 and 6 and movies in additional files 3, 4, 5 and 6. Mean quality score at 1.5 T (0.8 ± 0.4) was somewhat worse than at 3 T (0.6 ± 0.2), but the difference was not significant (p = 0.09). Mean quality score for 4D-SENSE (0.7 ± 0.2) did not differ from k-t BLAST (0.7 ± 0.4, p = 0.85).

## Discussion

The main finding of this study is that quantitative 4D flow accelerated with SENSE has good accuracy at 3 T and compares favourably to 1.5 T for intra-cardiac flow quantification. 4D flow accelerated with k-t BLAST underestimates flow velocities and thereby, in our opinion, yields too high bias for intra-cardiac quantitative in vivo use at the present time. The improved accuracy at 3 T may be explained by increased signal to noise ratio. For qualitative intra-cardiac 4D-flow visualization, however, 1.5 T and 3 T as well as SENSE or k-t BLAST can be used with similar quality.

### Previous validation studies on 4D-flow

There are several studies that have validated 4D-flow in the thoracic aorta with 2D-flow at 1.5 T [19-21]. However, there is a lack of validation of whole-heart 4D-flow studies and there is no data on the results at 3 T. Westenberg et al compared mitral and tricuspid flow quantified with 2D and 4D techniques using aortic flow as the reference at 1.5 T [13]. They used echo-planar imaging and a field of view limited to the atrioventricular plane to speed up the acquisition. Notably the accuracy in their study was higher for 4D-flow compared to 2D-flow for mitral and tricuspid flow compared to aortic flow. The bias of the present study using SENSE accelerated 4D-flow is similar to the findings of Westenberg et al. In a recent publication they showed comparable results with 4D flow in the atrioventricular plane with 2D flow of the MPA in healthy controls and also fair accuracy in 4D-flow measurements in patients with corrected tetralogy of Fallot [22]. Our results are also in line with the findings of Brix et al who compared 4D-flow to 2D-flow in the aorta and MPA at 1.5 T using the combination of echo-planar imaging and SENSE [23]. Using the results of the article their quantifications resulted in a bias calculated to -2 ± 16%. Eriksson et al used 4D-flow with SENSE in the left ventricle at 1.5 T and followed pathlines from the left ventriclular blood over the cardiac cycle to calculate stroke volume and compared the results with 2D-flow of the aorta [18]. Interestingly, the bias of our study is in the same range as the findings by Eriksson et al even if the method of analysis differ (-13 ± 11% calculated from the values presented in the article) [18].

Our results with k-t BLAST are in line with the in vitro measurements by Marshall [11] and the in vivo aortic quantifications by Stadlbauer et al [12]. Acceleration with k-t BLAST results in temporal blurring of the 4D flow as well as lower velocities mainly affecting systole and thereby a lower stroke volume. Our results show that there is a role for k-t BLAST for non-quantitative purposes, e.g. visualizing flow patterns, and the almost 50% reduction in acquisition times compared to the SENSE approach is especially important for patient studies.

Radial acquisitions with VIPR [24] to accelerate 4D-flow has recently been used in renal [25] and cerebral [26] arteries with encouraging results. The scanning times are reduced with VIPR and the technique is promising. Therefore, validation studies of VIPR for intra-cardiac applications are needed.

Our findings support the use of 3 T for intracardiac 4D-flow quantifications with high correlation and low bias compared to 2D-flow. The rather high bias in the present study suggests that k-t BLAST cannot be used for 4D-flow quantifications at the present time, especially at 1.5 T. However, for qualitative visualization purposes, acceleration with SENSE and k-t BLAST as well as acquisitions with 1.5 T and 3 T work equally well.

### Applications of 4D-flow

The possibilities of 4D-flow were recently reviewed in this journal [7]. Two examples of recent pathophysiological findings with this technique are the role of flow dynamics in the formation of aortic aneurysm in bicuspid aortic valves [27] and the impact of backward thoracic aortic flow in cerebral embolism originating from aortic plaques distal to the origin of the left common carotid artery [28]. Intra-cardiac applications of 4D-flow include quantification of valvular regurgitation [22] and diastolic properties of the left ventricle [29]. Bolger et al showed the proportions of stroke volume that leaves the LV at the same heart beat and the proportion that is ejected at the next heart beat [30] and Eriksson et al recently showed the advantageous position of the direct proportion of the stroke volume close to the LV outflow tract, which may have impact on pumping efficiency [31].

It is well known that too poor temporal resolution yields too low stroke volumes because of underestimation of peak flows. In this study the acquired temporal resolution was similar for SENSE and k-t BLAST (≈ 50 ms) but peak flows and stroke volumes were still lower with k-t BLAST. This suggests that the results are explained by inherent differences in the reconstruction process and not by differences in temporal resolution.

### Limitations

This study is limited by its small number of subjects but the results from 1.5 T with parallel imaging as well as k-t BLAST are in line with previous studies which indicate that the results would not differ with larger number of subjects.

SNR measurements were only performed in k-t BLAST images as SNR measurements in SENSE images are challenging and affected by many factors, e.g. spatial dependence of noise amplification, and the properties of the receiver coils [32,33]. k-t SENSE has been shown to be more accurate for 2D-flow quantifications compared to k-t BLAST [9] but k-t SENSE was not available at our scanner until the time of acquisition of the last subjects. This study did not evaluate different reconstruction settings for SENSE or k-t BLAST but rather used previously described parameters. Future studies could further investigate different reconstruction parameters varying acceleration, amount of training data and regularization settings to obtain the most accurate 4D-flow sequence.

k-t BLAST was performed with prospective ECG-triggering which results in lack of data during late diastole and atrial contraction. This is not optimal for flow quantification and is especially important for patients with valvular insufficiencies. Methods for retrospective k-t BLAST flow measurement has been described, however to our knowledge this has not yet been implemented for 4D-flow [34,35].

## Conclusions

Quantitative analysis of flow from 4D-PC-MRI on 3 T accelerated with parallel imaging has good accuracy and compares favourably with 1.5 T. Speed up with k-t BLAST for quantitative 4D-PC-MRI underestimate stroke volumes and peak flows and therefore yield too high bias for intra-cardiac quantitative in vivo use at the present time. 4D-flow visualization can be performed equally well at 1.5 T and 3 T, and SENSE and k-t BLAST show similar results.

## Competing interests

KMB is an employee of Philips Healthcare.

## Authors' contributions

MC, JT, MK and KMB carried out the data collection and analysis. EH and JT developed software for analysis. EH and FS participated in the design of the study. MC performed the statistical analysis. MC and HA conceived of the study, and participated in its design and coordination and drafted the manuscript. All authors revised the manuscript during its preparation and have read and approved the final manuscript.

## Supplementary Material

Additional File 1 **Flow curves versus time of six subjects at 3 T**. Click here for file

Additional File 2 **Flow curves versus time of six subjects at 1.5 T**. Click here for file

Additional File 3 **Animation showing typical 4D-flow visualization at 1.5 T, 4D-SENSE. Particle traces are coloured red in the left side and blue in the right side of the heart**. Click here for file

Additional File 4 **Animation showing typical 4D-flow visualization at 1.5 T, 4D k-t BLAST**. Particle traces are coloured red in the left side and blue in the right side of the heart. Click here for file

Additional File 5 **Animation showing typical 4D-flow visualization at 3 T, 4D-SENSE**. Particle traces are coloured red in the left side and blue in the right side of the heart. Click here for file

Additional File 6 **Animation showing typical 4D-flow visualization at 3 T, 4D k-t BLAST**. Particle traces are coloured red in the left side and blue in the right side of the heart. Click here for file
