# Supplementary figures and images for: Quantification and visualization of cardiovascular 4D velocity mapping accelerated with parallel imaging or k-t BLAST: head to head comparison and validation at 1.5 T and 3 T
Source: J Cardiovasc Magn Reson. 2011 Oct 4;13(1):55. doi: 10.1186/1532-429X-13-55 (PMC3213199; doi:10.1186/1532-429X-13-55)

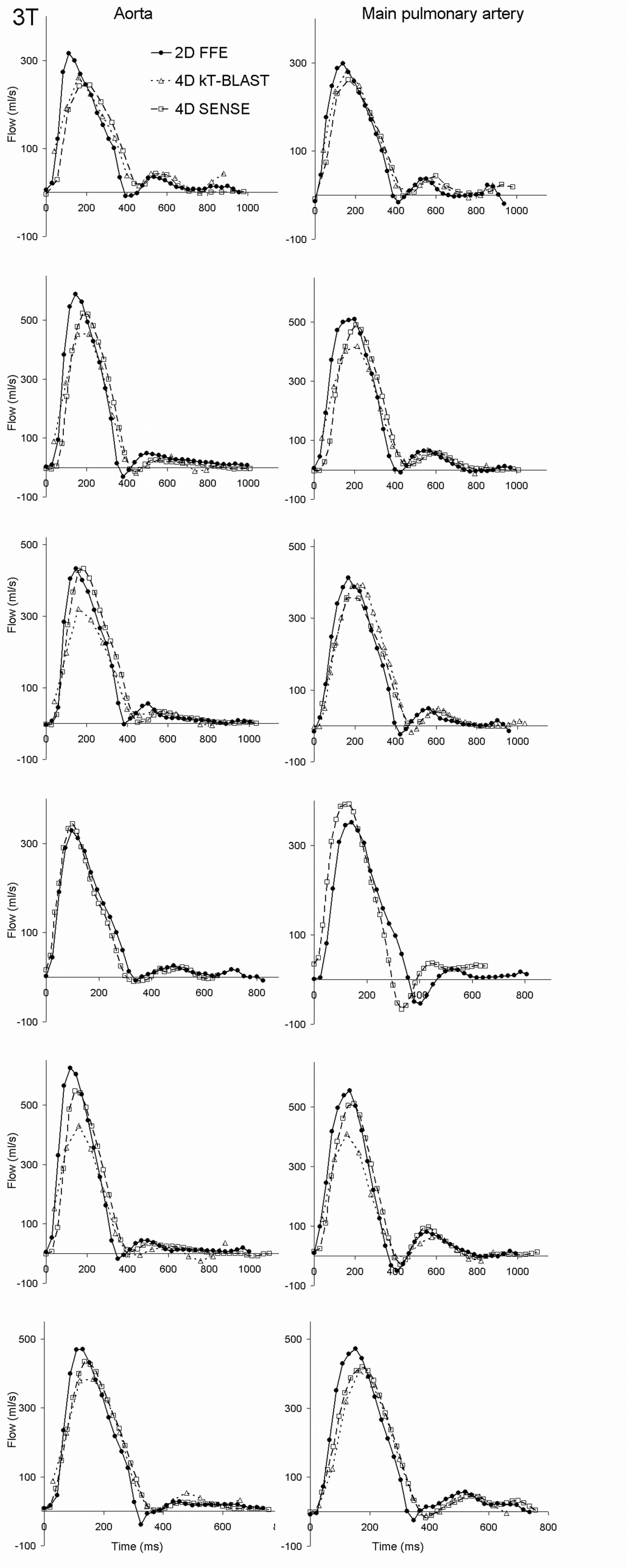

Supplement: Additional File 1 — Flow curves versus time of six subjects at 3 T. [file 1532-429X-13-55-S1.JPEG]

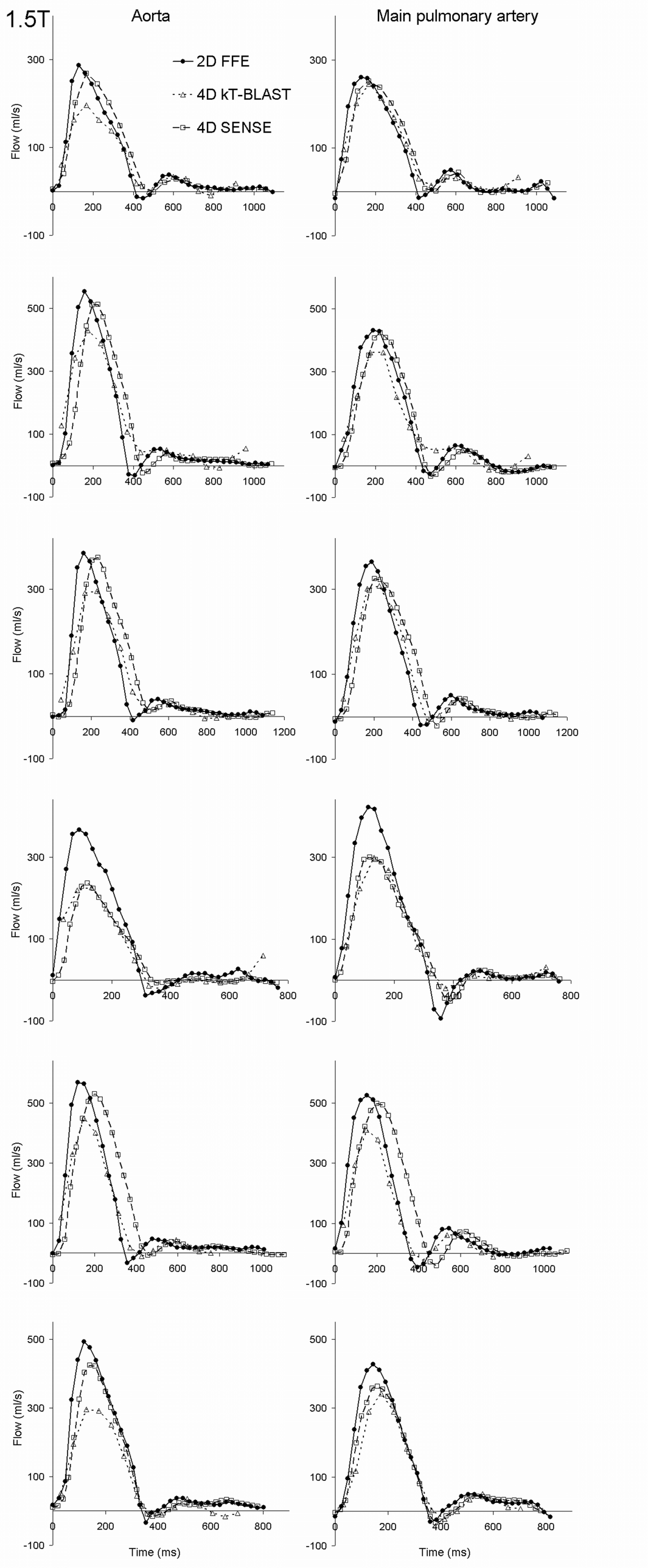

Supplement: Additional File 2 — Flow curves versus time of six subjects at 1.5 T. [file 1532-429X-13-55-S2.JPEG]
